# Cardiac Sympathetic Hyperactivity of Lung Cancer-Associated Harlequin Syndrome

**DOI:** 10.31662/jmaj.2018-0065

**Published:** 2019-07-08

**Authors:** Yuji Tanaka, Kazuo Satomi

**Affiliations:** 1 Department of Neurology, Gifu Municipal Hospital Gifu City, Gifu, Japan

**Keywords:** Cardiac sympathetic hyperactivity, Harlequin syndrome, Lung cancer, [^123^I]-metaiodobenzylguanidine scintigraphy, Metastatic tumor

A 79-year-old man with lung cancer, who was receiving chemotherapy, developed left-sided flushing and excessive sweating on his face, shoulder, and upper limb in response to exercise. Neurological examinations showed hypohidrosis on the right side of his face. He developed hypertension with tachycardia. Magnetic resonance imaging of the thoracic spine showed tumor invasion in the intervertebral foramen (Th2-3) (Figure 1). He was diagnosed with metastatic lung cancer that caused symptomatic harlequin syndrome by impairing the preganglionic sympathetic neuron (Th2-3). Cardiac [^123^I]-metaiodobenzylguanidine scintigraphy showed a normal early-phase heart-to-mediastinum ratio (2.50; reference range, 1.9-3.0) and an elevated washout rate (37.25%; reference range, 0%-34%) (Figure 2).

**Figure 1.**
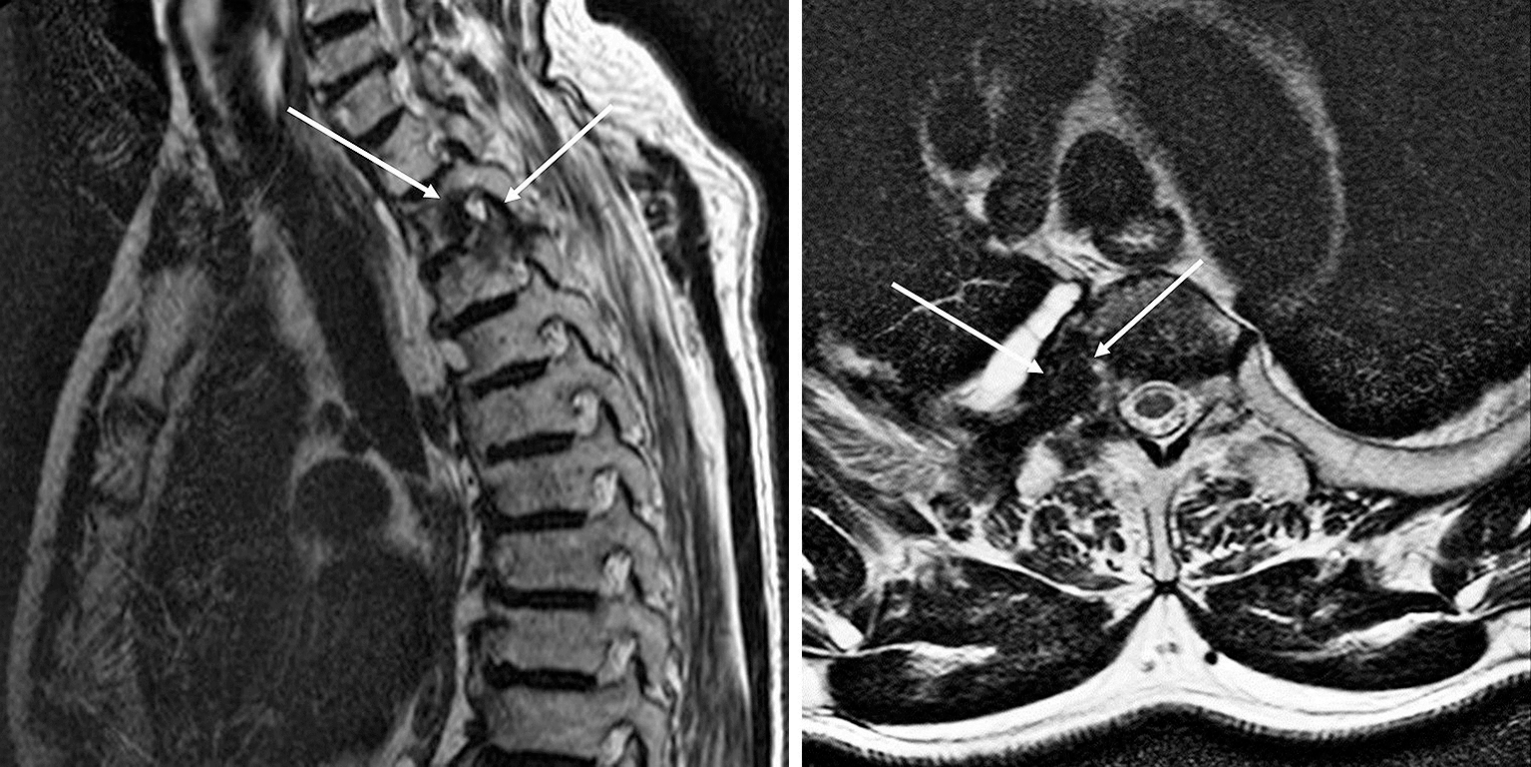
Magnetic resonance imaging of the thoracic spine showed tumor invasion in the intervertebral foramen.

**Figure 2.**
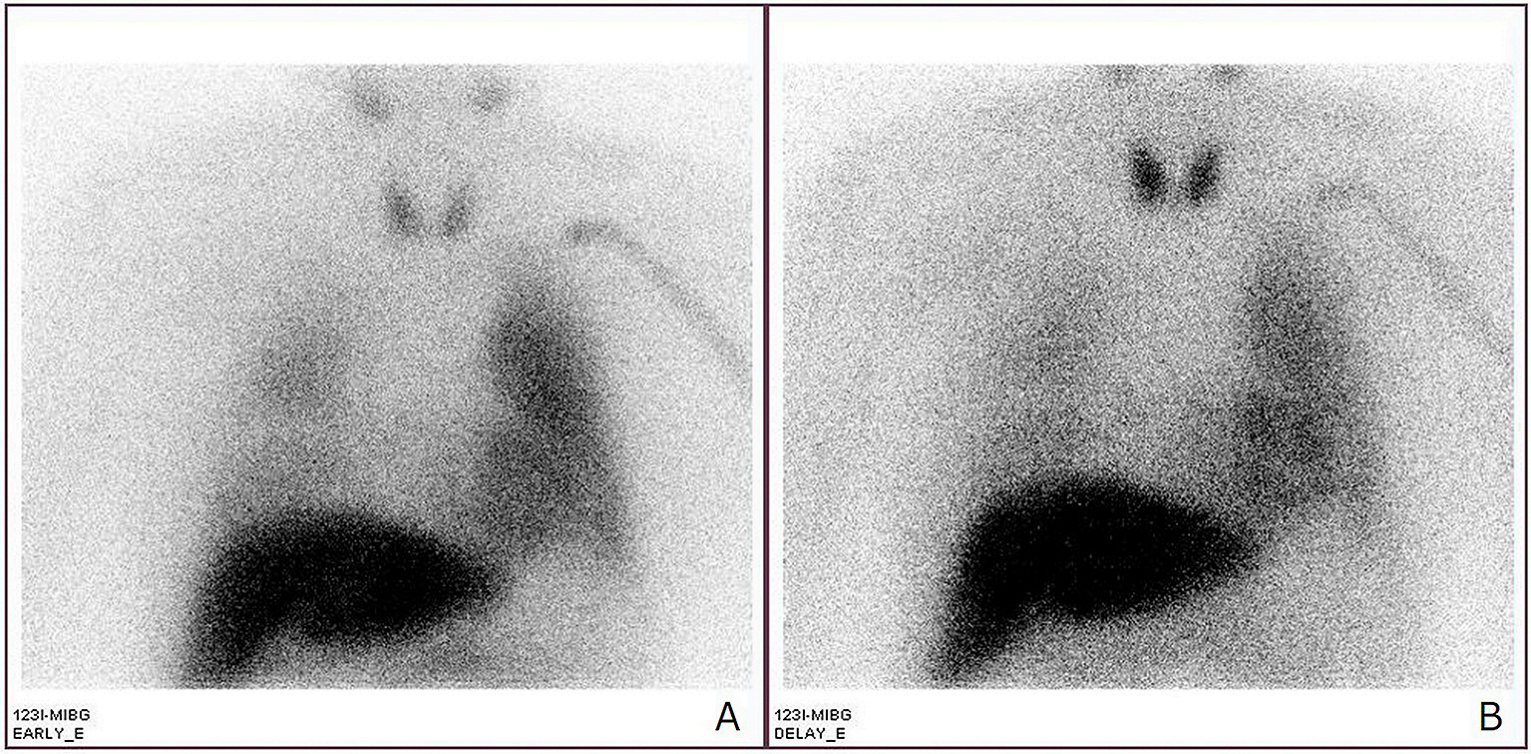
Cardiac [^123^I]-metaiodobenzylguanidine scintigraphy showed a normal early-phase heart-to-mediastinum ratio (2.50; reference range, 1.9-3.0) and an elevated washout rate (37.25%; reference range, 0%-34%) [(A) Early phase, (B) Delayed phase].

Although no report has described the cardiac sympathetic function of harlequin syndrome, our patient likely presented with cardiac sympathetic hyperactivity, as indicated by the increased washout rate ^(1), (2)^. This case showed two important issues: harlequin syndrome may be caused by tumors and patients with harlequin syndrome may exhibit cardiac sympathetic hyperactivity.

## Article Information

### Conflicts of Interest

None

### Author Contributions

YT wrote the manuscript. KS supervised all procedures.

### Approval by Institutional Review Board (IRB)

This manuscript is a case report.

### Informed Consent

Informed consent was obtained from the patient.
